# The Development and Validation of an Index to Evaluate the Quality of Work Life: A Sociolaboral Perspective Related to Nursing Staff

**DOI:** 10.7759/cureus.106150

**Published:** 2026-03-30

**Authors:** Miguel Angel Martínez-Hernandez, Lino Palacios-Cruz, Rodolfo Rivas-Ruiz, Juan Osvaldo Talavera-Piña, Victor Hugo Borja-Aburto

**Affiliations:** 1 Medical Education and Research Department, American British Cowdray Medical Center, Mexico City, MEX; 2 Clinical Epidemiology Department, National Institute of Psychiatry “Ramón de la Fuente Muñiz”, Mexico City, MEX; 3 Health Research Coordination, Mexican Social Security Institute, Mexico City, MEX; 4 Epidemiology and Public Health, Federal Commission for Protection Against Health Risks, Mexico City, MEX

**Keywords:** conjunctive consolidation, construct validity, development and validation, nursing staff wellbeing, nursing work environment, quality of work life, variable aggregation

## Abstract

Introduction

The development of indices enables the accurate identification of issues by consolidating the key factors associated with an outcome, which in turn allows for better prioritization of risks. Therefore, the purpose of creating indices is to generate information that supports evidence-based analysis, facilitating accurate decision-making and addressing risks through an understanding of the underlying components, thereby allowing decision-makers to develop prevention strategies and programs. The objective of this research was to develop and validate an index using the statistical technique of “conjunctive consolidation” to assess factors related to the quality of work life (QoWL) among nursing staff, providing a simple method that can be applied for specific predictions or decisions.

Methods

A methodological study was conducted using “conjunctive consolidation” analysis to develop an index of exposure factors associated with unsatisfactory QoWL among nursing staff at a tertiary care hospital. The index was created by consolidating the associated factors and assessing validity through convergence with constructs such as perceived institutional safety, musculoskeletal disorders, and mental health outcomes for each consolidation, as well as by evaluating the index’s performance using a receiver operating characteristic (ROC) curve.

Results

The consolidations used to develop the index were as follows: consolidation of psychosocial perception, comprising work alienation (odds ratio (OR): 3.8, 95% confidence interval (CI): 1.9-7.4) and reports of family aggression (OR: 3.5, 95% CI: 1.6-8.0); consolidation of exposure due to work activity, comprising forced movements (OR: 4.16, 95% CI: 2.13-8.15) and overexertion (OR: 4.43, 95% CI: 2.20-8.92); and consolidation of exposure due to work instruments, comprising exposure to drug preparation dust (OR: 3.61, 95% CI: 1.79-7.27) and exposure to radiation (OR: 3.66, 95% CI: 1.80-7.45). The diagnostic capacity of the index score was demonstrated by an area under the curve (AUC) of 0.79 (95% CI: 0.73-0.86, p < 0.001) on the ROC curve.

Conclusions

The development and validation of the index demonstrate that it can effectively measure QoWL among nursing staff and provide decision-makers with guidance on associated risks in hospital settings.

## Introduction

In the current healthcare landscape, the stability and operational effectiveness of healthcare systems rely on the health and professional continuity of the nursing staff. Nurses serve as the primary point of contact for patient care and encounter a unique combination of occupational stressors, including physical factors from exposure to work equipment, biological factors from exposure to pathogens, chemical factors from exposure to substances such as medications and residual anesthetic gases, ergonomic factors from repetitive tasks and sustained postures, and psychosocial factors arising from high job demands, low workplace social support, insufficient rest periods, and workplace violence, all of which are related to their work environment as well as broader social conditions [1,2].

Accordingly, quality of work life (QoWL) has emerged as a highly relevant, multidimensional construct that encompasses not only work satisfaction but also the overall well-being of employees within their professional environment [3,4]. However, the identification of risk factors associated with QoWL remains limited, as current public health approaches and policies often rely on subjective methods such as causal mapping, checklists, fault tree analysis, and Ishikawa diagrams. These approaches result in inadequate identification of risk factors and the effectiveness of interventions, leaving these workers as a vulnerable, at-risk group [5-8].

Even though several instruments, such as CVT-GOHISALO, define and assess QoWL, they primarily provide descriptive evaluations without considering the factors that influence it. These existing approaches are not necessarily multidimensional and seldom offer an objective metric that enables institutions to move from merely describing a problem to implementing targeted interventions [9]. For this reason, hospital organizations should have instruments and policies that guide when and how to evaluate risk factors, tailored to the context, objectives, and processes of the organization, thereby supporting initiatives that enhance resilience to crises and intervention strategies that improve the QoWL of their staff [10].

Certain evaluation methods, such as risk indices, allow for the assessment of both risk factors and overall health impact. They use statistical techniques that enable comparison of different factors, support the formulation of actions for a given level of risk, and measure their combined impact by aggregating multiple factors associated with the same outcome, resulting in a unified composite index of risk factors by category [11]. This study aimed to develop and validate an index using the statistical technique of conjunctive consolidation [12] to assess factors associated with QoWL among nursing staff in a tertiary care hospital in Mexico.

## Materials and methods

Study design and setting

The data for this methodological study were collected through a cross-sectional survey conducted among nursing staff at a tertiary-care hospital in Mexico City operated by the Mexican Social Security Institute (IMSS) from 2022 to 2024. Approval for the study was obtained by submitting the research protocol to the IMSS’s Electronic Registration System for Health Research Coordination (SIRELCIS), and upon approval, registration number R-2022-785-020 was granted by the National Committee for Scientific Research of the Mexican Social Security Institute. To establish the study's feasibility, the sample size was determined using the Lwanga-Lemeshow formula to assess associations between exposure factors and QoWL (p = 0.3, RR = 3.0, 0.2); additionally, the Freeman formula was applied [13], respecting the concept of “events per variable” [14]. The study included 353 nursing professionals.

Data collection

Data were collected prospectively by two clinical assistants from the Health Prevention and Promotion Service of the hospital’s Occupational Health Department. For this process, the clinical assistants actively invited nursing staff to participate; those who agreed were provided with an explanation of the study’s purpose and ethical considerations and asked to give their consent via a fillable online form accessible on their mobile phones, which redirected them to a webpage where they could review the consent at that time or later. This centralized data collection by specialized personnel from the Occupational Health Department ensured that the process was standardized and conducted in a safe, professional environment for all participants.

Regarding the independent factors (exposures), items from institutionally recognized occupational health records were included to ensure that the index could be harmonized with existing hospital data. The instruments used to collect data on social and occupational risk factors were based on a comprehensive medical and occupational history, including personal pathological, non-pathological, hereditary/family, and occupational history. Additionally, the AUDIT questionnaire was used to assess alcohol consumption, the Fagerström test to evaluate nicotine dependence, and the Inventory of Violence and Psychological Harassment at Work to assess workplace violence, all of which have been validated for the Mexican population.

The assessment of the dependent variable was based on the CVT-GOHISALO scale, which is validated for healthcare personnel. The scale includes 74 items across seven dimensions: institutional support for work, workplace safety, job integration, job satisfaction, well-being achieved through work, personal development, and management of free time. The complete set of instruments is referred to as the Epidemiological Survey of Quality of Life at Work (EPICAVT). Data from each questionnaire were exported to a database in IBM SPSS Statistics v27.0 (IBM Corp., Armonk, NY) [15]. McCall’s T-score was applied to dichotomize QoWL, converting raw scores into a standardized scale (mean: 50, standard deviation (SD): 10) to establish a comparative cutoff identifying the high-risk subgroup. Based on this standardization, a cutoff score of 60 was set to define “unsatisfactory QoWL.”

For the validated scales and instruments assessing independent factors, final scores were calculated, while variables from the medical and occupational history were categorized for subsequent analysis using the "conjunctive consolidation" technique.

Data analysis

The statistical method of "conjunctive consolidation" was used, a multivariate technique that orders the data into groups and allows the researcher to reflect on the variables' relevance by organizing the data into clusters [16]. The factors used to create the index were related to occupational and non-occupational exposure, and the index was developed and validated in three steps, which are detailed below:

Step 1: Generation of the Index

Data from a cross-sectional survey were used, together with a “multivariable categorical groups” approach. This approach sought to examine the bivariate predictive effect of each factor, subsequently generating aggregations of the variables. This step was carried out in two stages.

Stage 1 (step 1): Contingency tables were created to evaluate all joint tabulations between the different exposure factors and QoWL categories. Three criteria were used to assess the relevance of variables for aggregation in the index: (1) the adequacy of the data was assessed, verifying that there were at least 20 subjects for each subgroup and that the events per category of each variable were ≥10 subjects in the column of “unsatisfactory QoWL” [16]. (2) Inter-category gradients were verified; that is, the minimum acceptable percentage difference (gradient) between the clusters that reached the outcome of interest (unsatisfactory QoWL) was ≥10%; and similarly, the intrasystem gradient was evaluated, that is, the minimum percentage difference between the primary category and the minor category of each variable, considering the minimum acceptable value to be 10%. (3) The difference between satisfactory and unsatisfactory QoWL was analyzed for each exposure factor and the measures of association using Pearson's χ2 test.

Stage 2 (step 1): Two processes were carried out to aggregate the variables: (1) conjunction of variables: each exposure factor was paired with another, assessing the similarity between qualities of the variables, which generated more relevant gradients when aggregated than individually. The effects of two combined variables were calculated in a table format. (2) Consolidation of variables: the joint tabulations were evaluated for all pairs of variables to create a single composite variable of the two chosen exposure factors, and it was given a name according to the nature of the exposure factors that composed it. Each category of the consolidation of variables was called a “level”.

Step 2: Construction of the Final Index and Validity

Two processes were carried out in this regard:

(1) A binary logistic regression analysis was carried out between the consolidations and the QoWL, using the backward elimination technique, retaining the consolidations with higher goodness of fit, with face and content validity. Face validity was determined through the judgment of a panel of experts in occupational medicine and clinical epidemiology, who selected factors based on scientific evidence and the availability of data in the real-world hospital setting to verify the tool’s feasibility. The consolidations used are not merely aggregated, but clustered strategically to represent a biological or social gradient of exposure. Theoretically, these consolidations reflect the synergy of risk factors-the idea that the convergence of multiple stressors creates a pathogenic state that impacts the QoWL more significantly than each factor would in isolation. This method allows the index to maintain clinical common sense while ensuring statistical stability. Each consolidation that was included in the final model was subsequently validated through a convergence analysis to generate construct validity: the consolidation “psycho-environmental perception” was associated with the presence of diagnoses of damage to mental health, the consolidation “exposure due to work activity” with diagnoses of musculoskeletal disorders and finally the consolidation “exposure due to work instruments” with perception of safety provided by the institution.

(2) The association measures of the chosen model in the binary logistic regression were analyzed, and the rounded OR-1 value of the categories of each consolidation was used to generate the overall index score.

The final version of the index was designed, and the score of each of the consolidations generated was calculated to obtain the total index score for each nurse (total score in points). A ROC curve was constructed between the total index score and the QoWL level for two purposes: (1) to establish a cutoff point for the total index score associated with an unsatisfactory QoWL level, which was established using Youden's J index, by selecting the cutoff point at which the highest sensitivity and specificity are achieved; (2) to evaluate the discriminatory capacity of the index created, evaluating the area under the curve (AUC) of the overall score and each of the consolidations generated.

## Results

Step 1

Thirteen theoretical models of the factors associated with unsatisfactory QoWL were analyzed preliminarily, considering their purpose, comprehensibility, replicability, appropriate output scale, content, face validity, and ease of use. This demonstrates the index's diagnostic and predictive utility. The study included 353 nursing staff, of whom 83.3% were women, with a median age of 40 years, and 95.5% worked in a non-surgical department. The prevalence of unsatisfactory QoWL was 11.6%.

First, the intrasystem gradient was analyzed. This refers to the minimum acceptable percentage difference of ≥10% between subjects without and with the risk factor and unsatisfactory QoWL. The variable “reporting family aggression” had a gradient ranging from 9.8% to 27.8% (difference of 18%), “perception of alienation at work” from 7.8% to 24.1% (difference of 16.3%), “overstrain” from 5.8% to 21.5% (difference of 15.7%), “forced movements” from 6.8% to 23.3% (difference of 16.5%), “exposure to radiation” from 6% to 19% (difference of 13%), and “exposure to dust from the preparation of medicines” from 8.6% to 25.4% (difference of 16.8%), as well as “exposure to electrosurgical fumes”.

Because they did not meet the minimum sample size required for each cell (≥10 subjects in the numerator and ≥20 subjects in the denominator), the following variables were not included in any consolidation: inadequate eating habits, abuse by colleagues or boss, prolonged standing, and prolonged squatting (Table 1).

**Table 1:** Assessment of the adequacy of inter-category gradients, intra-system gradients, and bivariate analysis for an unsatisfactory QoWL (step 1, stage 1) ^*^Meets the minimum accepted inter-category and intra-system gradient criteria of 10%, as well as the minimum cell size required (10 subjects in the numerator and 20 subjects in the denominator) QoWL: quality of work life; OR: odds ratio; CI: confidence interval

Unsatisfactory QoWL, 11.6%					
Variable	Category	Unsatisfactory QoWL, n (%)	Intrasystem gradients	Non-adjusted OR (95% CI)	P-value
Marital status (partner at home)	a1: With partner	14/185 (7.6%)	a2-a1 = 8.5%	2.339 (1.181 – 4.630)	0.013
	a2: Without partner	27/168 (16.1%)			
Report of domestic violence^*^	b1: Never	31/317 (9.8%)	b2-b1 = 18%	3.548 (1.566 – 8.041)	0.001
	b2: Physical or verbal aggression	10/36 (27.8%)			
Incapacity due to occupational accidents or diseases in the last year	c1: No	30/297 (10.1%)	c2-c1 = 9.5%	2.176 (1.018 – 4.650)	0.041
	c2: Yes	11/56 (19.6%)			
Exposure to vibrations in the arm-hand system	d1: No	29/292 (9.9%)	d2-d1 = 9 .8%	2.221 (1.061 – 4.649)	0.031
	d2: Yes	12/61 (19.7%)			
Poor lighting in the working area^*^	e1: No	25/276 (9.1%)	e2-e1 = 11.7%	2.633 (1.325 – 5.235)	0.005
	e2: Yes	16/77 (20.8%)			
Exposure to extreme temperatures	f1: No	22/238 (9.2%)	f2-f1 = 7.3%	1.943 (1.005 – 3.757)	0.045
	f2: Yes	19/115 (16.5%)			
Exposure to radiation^*^	g1: No	12/200 (6.0%)	g2gf1 = 13%	3.664 (1.802 – 7.452)	< 0.001
	g2: Yes	29/153 (19%)			
Exposure to dust from drug preparation^*^	h1: No	25/290 (8.6%)	h2-h1 = 16.8%	3.609 (1.791 – 7.267)	< 0.001
	h2: Yes	16/63 (25.4%)			
Exposure to electrosurgical fumes^*^	i1: No	30/306 (9.8%)	i2-i1 = 13.6%	2.811 (1.297 – 6.091)	0.007
	i2: Yes	11/47 (23.4%)			
Exposure to medicinal gases and vapors	j1: No	26/274 (9.5%)	j2-j1 = 9.5%	2.236 (1.119 – 4.468)	0.020
	j2: Yes	15/79 (19%)			
Exposure to disinfection liquids and solvents	k1: No	26/273 (9.5%)	k2-k1 = 9.3%	2.192 (1.098 – 4.378)	0.027
	k2: Yes	15/80 (18.8%)			
Perception of work stress^*^	L1: No	21/270 (7.8%)	L2-L1 = 16.3%	3.764 (1.923 – 7.370)	< 0.001
	L2: Yes	20/83 (24.1%)			
Forced postures	m1: No	22/248 (8.9%)	m2-m1 = 9.2%	2.270 (1.170 – 4.401)	0.013
	m2: Yes	19/105 (18.1%)			
Physical overexertion^*^	n1: No	13/223 (5.8%)	n2-n1 = 15.7%	4.434 (2.204 – 8.921)	< 0.001
	n2: Yes	28/130 (21.5%)			
Forced movements^*^	o1: No	17/250 (6.8%)	o2-o1 = 16.5%	4.164 (2.127 – 8.151)	< 0.001
	o2: Yes	24/103 (23.3%)			
Stretching due to inadequate work area dimensions	p1: No	22/253 (8.7%)	p2-p1 = 10.3%	2.463 (1.268 – 4.784)	0.006
	p2: Yes	19/100 (19%)			
Poor workspace layout^*^	q1: No	17/229 (7.4%)	q2-q1 = 12%	2.993 (1.539 – 5.821)	< 0.001
	q2: Yes	24/124 (19.4%)			
Dietary habits	l1: Adequate	5/105 (4.8%)	l2-l1 = 9.7%	3.396 (1.294 – 8.916)	0.009
	l2: Inadequate	36/248 (14.5%)			
Mistreatment by coworkers or boss	m1: No	34/333 (10.2%)	m2-m1 = 24.8%	4.735 (1.768 – 12.679)	< 0.001
	m2: Yes	7/20 (35%)			
Prolonged standing at work	n1: No	6/115 (5.2%)	n2-n1 = 9.5%	3.132 (1.278 – 7.679)	0.008
	n1: Yes	35/238 (14.7%)			
Prolonged squatting at work	o1: No	34/332 (10.2%)	o2-o1 = 23.1%	4.382 (1.654 – 11.609)	0.002
	o2: Yes	7/21 (33.3%)			

Secondly, conjunctions were generated, which involve pairing variables to form a new composite variable, and consolidations, which involve grouping two or more categories to create a new variable with defined levels of outcome presentation (Table 2).

**Table 2:** Generation of the index using the “conjunctive consolidation” technique (step 1, stage 2) Note: The variable of exposure to electrosurgical fumes was not added to any other variable because it is only present in surgical-area personnel QoWL: quality of work life

1: Conjunctive consolidation of reports of domestic violence – perception of work stress for unsatisfactory QoWL				Perception of work stress		
				No, n (%)	Yes, n (%)	The gradient of reports of domestic violence, 9.8% to 27.8%, n (%)
	Report of domestic violence		Never	17/250 (6.8%), level I	14/67 (20.9%), level II	31/317 (9.8%)
			Physical or verbal aggression	4/20 (20%), level II	6/16 (37.5%), level III	10/36 (27.8%)
	Gradient of perception of work stress: 7.8% to 24.1%			21/270 (7.8%)	20/83 (24.1%)	41/353
	Generation of predictive consolidation of “psycho-environmental perception” with three levels: 7%, 21%, and 38%. The consolidated internal cells are groups of composite categories					
2: Conjunctive consolidation of physical overexertion – forced movements for unsatisfactory QoWL				Forced movements		
				No, n (%)	Yes, n (%)	Gradient of physical overexertion: 5.8% to 21.5%, n (%)
	Physical overexertion		No	10/210 (4.7%), level I	3/13 (23%), level II	13/223 (5.8%)
			Yes	7/40 (17.5%), level II	21/90 (23%), level II	28/130 (21.5%)
	Gradient of forced movements: 6.8% to 23.3%			17/250 (6.8%)	24/103 (23.3%)	41/353 (11.6%)
	Generation of predictive consolidation of “exposure due to work activity” with two levels: 5% and 22%. The consolidated internal cells are composite category groups					
3: Conjunctive consolidation of exposure to radiation – exposure to dust from drug preparation for unsatisfactory QoWL				Exposure to dust from drug preparation		
				No, n (%)	Yes, n (%)	Gradient of exposure to radiation 6% to 19%, n (%)
	Exposure to radiation	No		6/183 (3.2%), level I	6/17 (35.2%) level II	12/200 (6.0%)
		Yes		19/107 (17.7%), level II	10/46 (21.7%), level II	29/153 (19%)
	Gradient of exposure to dust from drug preparation: 8.6% to 25.4%			25/290 (8.6%)	16/63 (25.4%)	41/353
	Generation of predictive consolidation of “exposure by work instruments” with two levels: 3% and 35%. Consolidated internal cells are groups of composite categories					
4: Conjunctive consolidation of poor lighting in the working area – poor workspace layout for unsatisfactory QoWL				Poor workspace layout		
				No, n (%)	Yes, n (%)	Gradient of poor lighting in working area: 9.1% to 20.8%
	Poor lighting in the working area	No		12/193 (6.2%), level I	13/83 (15.6%), level II	25/276 (9.1%)
		Si		5/36 (13.8%), level II	11/41 (26.8%), level III	16/77 (20.8%)
	Gradient of poor workspace layout 7.4% to 19.4%			17/229 (7.4%)	24/124 (19.4%)	41/353 (11.6%)
	*Generation of predictive consolidation of “exposure by the workplace” with three levels: 6%, 15%, and 27%. The consolidated internal cells are groups of composite categories					

The conjunctions were generated for the variables that met the criterion of the intrasystem gradient and their nature. Finally, the consolidations obtained were as follows: (1) psycho-environmental perception: report of domestic violence and perception of work stress (prediction with three levels: with a gradient from 7% to 38%), (2) exposure due to work activity: physical overexertion and forced movements (prediction with two levels: with a gradient from 5% to 22%), (3) exposure by work instruments: exposure to radiation and exposure to dust from drug preparation (prediction with two levels: with a gradient of 3% to 21%), (4) exposure by the workplace: poor lighting in working area and poor workspace layout (prediction with three levels: with a gradient of 6% to 27). The variable exposure to electrosurgical fumes was not consolidated with any other variable, as it only affects personnel in surgical areas (Table 2).

Step 2

Multiple logistic regression analysis with backward selection was performed on the four consolidations obtained. The third model was selected, retaining the psycho-environmental perception consolidation (level II OR: 2.49; 95% CI: 1.17-5.31 and level III OR: 6.67; 95% CI: 1.65-19.47); exposure due to work activity consolidation (level II OR: 2.75; 95% CI: 1.18-6.45) and of exposure by work instruments consolidation (level II OR: 5.44; 95% CI: 2.15-3.71) (Table 3). Likewise, a convergent validity analysis of the final consolidations was performed, where “psycho-environmental perception” was associated with the presence of mental health disorders (level II OR: 2.37; 95% CI: 1.10 - 5.10 and level III OR: 10. 48; 95% CI: 3.47-31.60); “exposure due to work activity” was associated with diagnoses of musculoskeletal disorders (level II OR: 1.93; 95% CI: 1.12-3.33); and finally, “exposure by work instruments” was associated with the perception of safety provided by the institution (level II OR: 3.60; 95% CI: 2.10-6.18) (Table 3).

**Table 3:** Logistic regression analysis (backward selection) for the creation of the final index and construct validity analysis (step 2) ^*^Only the model 3 of the multiple logistic regression performed in step 2 is shown Model 1 R^2^ = 0.255; variable “exposure to electrosurgical fumes” (OR: 1.130, 95% CI: 0.472–2.705) was removed. Model 2 R^2^ = 0.255; consolidation “exposure by the workplace” (level II OR: 1.109, 95% CI: 0.439–2.806, and level III OR: 1.865, 95% CI: 0.608 – 5.724) was removed QoWL: quality of work life; OR: odds ratio; CI: confidence interval

	Model 3 (R^2 ^= 0.248)	Adjusted OR (95% CI)^*^	P-value	Construct validity	Non-adjusted OR (95% CI)	P-value
Psycho-environmental perception	Level I	Ref.		Convergence with mental health disorders	Ref.	
	Level II	2.490 (1.168-5.305)	0.018		2.366 (1.098-5.102)	0.028
	Level III	6.674 (1.654-19.466)	0.006		10.477 (3.474-31.595)	< 0.0001
Exposure due to work activity	Level I	Ref.		Convergence with diagnoses of musculoskeletal disorders	Ref.	
	Level II	2.752 (1.175-6.446)	0.020		1.927 (1.117-3.325)	0.018
Exposure to work instruments	Level I	Ref.		Convergence with the perception of safety provided by the institution	Ref.	
	Level II	5.435 (2.154-13.713)	< 0.0001		3.602 (2.099-6.182)	< 0.0001

**Figure 1:**
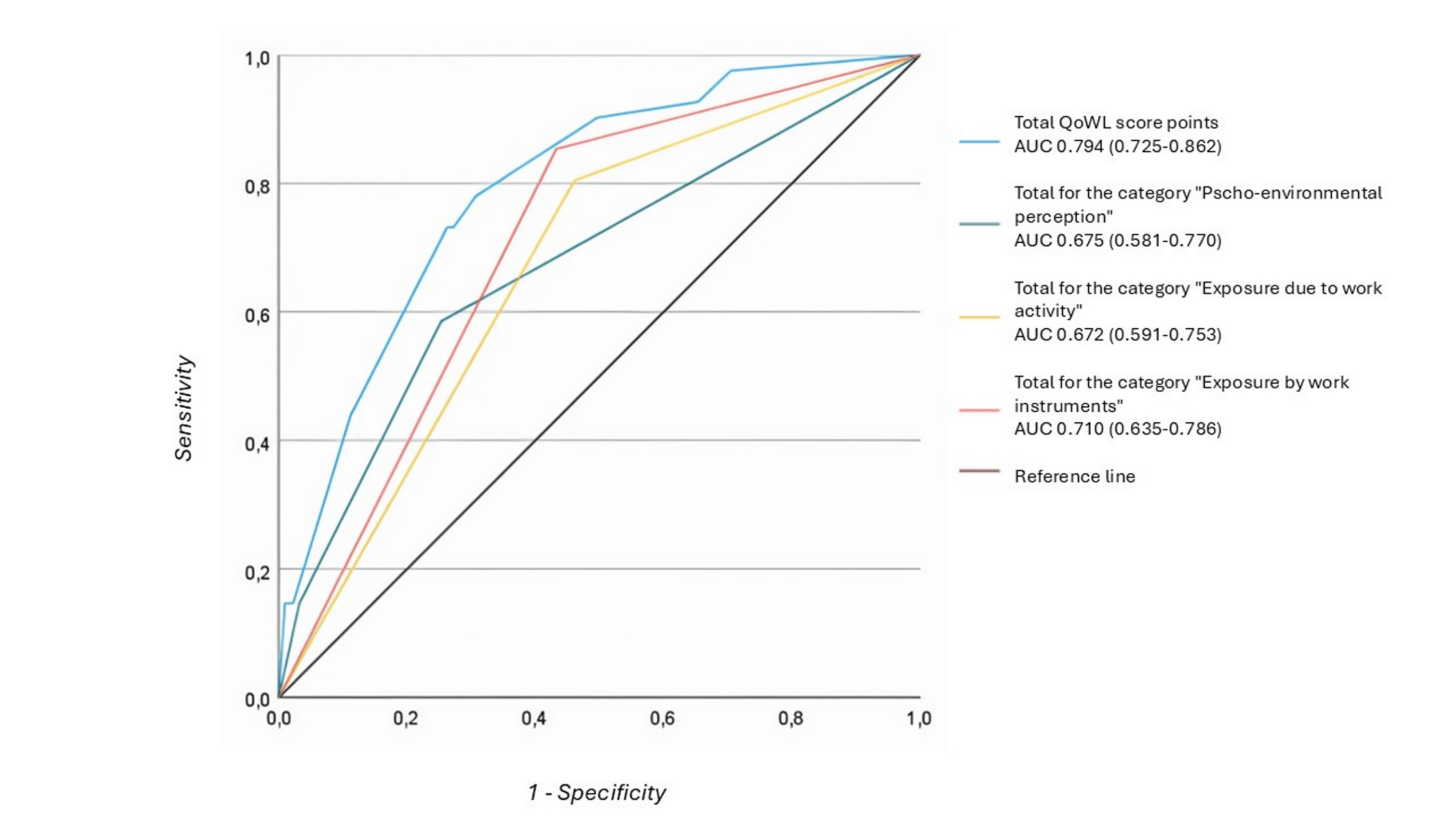
Diagnostic performance analysis: final index score and unsatisfactory QoWL QoWL: quality of work life; AUC: area under the curve

Finally, using Youden's J index, a cutoff point (4 points) was established for the overall score (14 points) that predicts unsatisfactory QoWL. To evaluate the diagnostic performance of the index, both the final index score and the three components that comprise it were evaluated for the prediction of unsatisfactory QoWL, obtaining an area under the curve of the overall score of 0.794 (0.725-0.862, p < 0.001) (Figure 1). The final index is shown in the Appendices.

## Discussion

In the present study, an index was developed and validated to evaluate risk factors associated with unsatisfactory QoWL among nursing staff; it demonstrated adequate clinimetric properties for face, construct, and criterion validity. Compared with our index, traditional scales only evaluate risk factors or QoWL separately, preventing workers from understanding the causes that affect their QoWL. To develop the index, a descriptive analysis of the nursing staff was first conducted, which revealed that 11.6% of the nursing staff had an unsatisfactory QoWL. Although this figure may not seem alarming, it should be noted that 41 nurses were affected and required individual attention, as noted by the International Labour Organization [11,17,18].

Based on our findings and in line with the models proposed by Turcotte and Patlan, certain non-work-related factors affect QoWL; therefore, we decided to include them in the present index so that workplaces can incorporate them as variables to be recognized, addressed, and managed through appropriate support measures [19-22]. On the other hand, the work-related factors that were associated with unsatisfactory QoWL included incapacity due to occupational accidents or diseases in the past year, mistreatment by coworkers or supervisors, and perceived work stress, which are probably related to workload, long working hours, poor social and interpersonal relationships, and low personal development, as well as a negative perception of the workplace. These factors have a direct impact on the work team, absenteeism, and work-related illnesses, as proposed by Henríquez-Figueroa and collaborators [23].

Other occupational risk factors that stood out in our study were physical risk factors such as poor lighting in working areas; exposure to extreme temperatures (cold and heat) in the sterilization center, operating rooms, and cold chain; as well as vibrations affecting the arm-hand system generated by work instruments, exposure to radiation from work-related equipment, dust generated in the preparation of drugs, exposure to electrosurgical fumes, medicinal gases and vapors, and disinfectants and solvents [24,25]. Similarly, risk factors related to work activity, such as prolonged standing and squatting, forced postures, physical overexertion due to work activity, forced movements, overstretching due to inadequate workspace dimensions, and poor workspace layout, were observed [26,27].

Conjunctive consolidation analysis allows us to examine how certain combinations of exposures amplify the risk of an unsatisfactory QoWL, revealing synergistic effects. Compared to the international literature, our results confirm that the interaction of physical demands with a negative psycho-environmental setting contributes to a significantly higher risk compared to the presence of factors in isolation. This distinction from external evidence confirms the interpretive merit of our results. Regarding the construction of the index, it is important to mention that this study was limited to certain components of socio-occupational exposure, which means that other factors that could have impacted QoWL were not included in the final index. While this likely limits the range of the analysis due to the omission of these variables, the existing index was designed to provide a high-impact, readily available list of predictive factors in a hospital context to ensure the index's immediate applicability.

The first factors to be excluded from the initial analysis evaluating the inter-category and intra-system gradients were poor dietary habits, mistreatment by colleagues or boss, prolonged standing, and prolonged squatting, because the requirement of ≥10 subjects in the numerator and ≥ 20 subjects in the denominator for the QoWL ratio was not met. The following factors were also excluded: marital status (partner at home), incapacity due to occupational risk in the last year, exposure to arm-hand system vibrations, extreme temperatures, medicinal gases and vapors, disinfection liquids and solvents, forced postures, and stretching due to inadequate work area dimensions, because they did not meet the criteria of the inter-category and intra-system gradients of at least a 10% difference between the categories of each dichotomous variable [16,28].

While the variable “dietary habits” was included as a predictor in the bivariate phase, it was excluded from the final index based on a criterion of institutional feasibility that favored those domains in which workplaces have direct capacity for intervention and modification [11]. In addition, exposure to electrosurgical fumes was initially eliminated for two reasons. The first was based on clinical criteria, since most nursing staff were in hospital areas (administrative, admissions, emergency, and intensive care), accounting for 99.5%, and a minority in the surgical area, accounting for 4.5%. The second criterion was statistical, because this variable lost its statistical significance in the first multivariable model.

Likewise, when carrying out the multivariate analysis, it was decided not to include in the final index the consolidation of “exposure in the workplace” (consisting of “poor lighting in work areas and poor workspace layout”), as this consolidation did not explain more variance than that observed in model 3. Additionally, the variables that formed this consolidation cannot be impacted immediately, as they depend on institutional policy reforms and funding [29]. It is essential to note that, although these factors have been excluded from the final index, they should still be considered in occupational health and safety programs, since they must be addressed due to their association with QoWL risk among nursing staff, and do not exempt us from applying preventive and corrective actions. In the future, the tool should be modified to include a broader spectrum of indicators to further enhance its comprehensive explanatory power.

It is crucial to bear in mind that the factors affecting each workplace, and each worker, vary across places and over time for the recognition, evaluation, and control of the working environment [30]. Therefore, we must emphasize that this index should be used by analyzing the particular characteristics of each hospital as a whole to define and prioritize additional factors that affect the QoWL of the nursing staff of each institution. While internal and construct validity are strong, we acknowledge that the results require external validation. The next steps will be to test this index at different levels of care and in both public and private hospital settings, thereby establishing its sensitivity and specificity in populations with different organizational structures.

Finally, a limitation of this study was that the sample size for the unsatisfactory QoWL ratio did not meet the required threshold for certain factors; therefore, we recommend that future studies use larger data sets, which could be achieved through nursing staff censuses or longitudinal evaluations. This would allow the criteria for the minimum number of subjects required within each subgroup to be met, as well as the required number of events per category for each variable, thus enabling the inclusion of all statistically significant factors, which would refine the index proposed here and provide a roadmap for future research.

## Conclusions

Our index is a new process that integrates the evaluation of QoWL and the exposure factors that affect it, which is essential for understanding the overall context of nursing staff work and for obtaining information to support decision-making and actions within the hospital setting. Therefore, it represents a simple method that can be applied for predictions and specific decision-making, in which the consolidated groups do not contain mixtures of arbitrarily combined and isolated categories, which makes it a robust and easy-to-apply method.

## Appendices

**Table 4:** Risk index for unsatisfactory quality QoWL: quality of work life

Read the indications and answer the corresponding sections, according to your role within the hospital center					
Step 1: Area to be filled by the nursing staff member. Instructions: assess the factors to which you are exposed in your work area and circle 1 if you are exposed to those factors and 0 if you are not exposed to the factors listed. At the end, add up the points for each item					
Psycho-environmental perception				No	Yes
Perception of work stress				0	1
Domestic violence				0	1
Total for this category				^(A)^	
Exposure due to work activity				No	Yes
Forced movements				0	1
Physical overexertion				0	1
Total for this category				^(B)^	
Exposure by work instruments				No	Yes
Exposure to dust from drug preparation				0	1
Exposure to radiation at your workplace				0	1
Total for this category				^(C)^	
Step 2: Area to be filled by the nursing staff member. Instructions: based on the score obtained from the sum of each item in Step 1, select the corresponding score and add up the points; this final score will allow you to evaluate your quality of work life (QoWL)					
	Total for the given category from step 1	Total QoWL score points	QoWL evaluation score		
Psycho-environmental perception^(A)^	0	0	^**^Satisfactory QoWL: 4 points or less. ^**^Unsatisfactory QoWL: 5 or more points		
	1	1			
	2	6			
Exposure due to work activity^(B)^	0	0			
	≥ 1	3			
Exposure by work instruments^(C)^	0	0			
	≥ 1	4			
Final index score^**^		**			
Step 3: Area to be filled in by the occupational health service personnel. Instructions: based on the total for the given category from step 1, circle the risk priority level that corresponds to each item and take the appropriate action					
	Total for the given category from step 1	Risk prioritization level	Prioritization actions		
Psycho-environmental perception^(A)^	0	I	Level I: continue with the current approach and evaluate according to local guidelines		
	1	II	Levels II and III: referral to workplace mental health services and/or change in working practices		
	2	III			
Exposure due to work activity^(B)^	0	I	Level I: continue with the current approach and evaluate according to local guidelines		
	≥ 1	II	Level II: (1) provide personal protective equipment and/or isolate the risk factor; (2) replace the risk factor and/or physically remove the risk factor		
Exposure by work instruments^(C)^	0	I	Level I: continue with the current approach and evaluate according to local guidelines		
	≥ 1	II	Level II: (1) provide personal protective equipment and/or isolate the risk factor; (2) replace the risk factor and/or physically remove the risk factor		
